# Changes in respiratory and non-respiratory symptoms in occupants of a large office building over a period of moisture damage remediation attempts

**DOI:** 10.1371/journal.pone.0191165

**Published:** 2018-01-11

**Authors:** Ju-Hyeong Park, Sook Ja Cho, Sandra K. White, Jean M. Cox-Ganser

**Affiliations:** Respiratory Health Division, National Institute for Occupational Safety and Health, Morgantown, West Virginia, United States of America; Universite de Bretagne Occidentale, FRANCE

## Abstract

There is limited information on the natural history of building occupants’ health in relation to attempts to remediate moisture damage. We examined changes in respiratory and non-respiratory symptoms in 1,175 office building occupants over seven years with multiple remediation attempts. During each of four surveys, we categorized participants using a severity score: 0 = asymptomatic; 1 = mild, symptomatic in the last 12 months, but not frequently in the last 4 weeks; 2 = severe, symptomatic at least once weekly in the last 4 weeks. Building-related symptoms were defined as improving away from the building. We used random intercept models adjusted for demographics, smoking, building tenure, and microbial exposures to estimate temporal changes in the odds of building-related symptoms or severity scores independent of the effect of microbial exposures. Trend analyses of combined mild/severe symptoms showed no changes in the odds of respiratory symptoms but significant improvement in non-respiratory symptoms over time. Separate analyses showed increases in the odds of severe respiratory symptoms (odds ratio/year = 1.15‒1.16, p-values<0.05) and severity scores (0.02/year, p-values<0.05) for wheezing and shortness of breath on exertion, due to worsening of participants in the mild symptom group. For non-respiratory symptoms, we found no changes in the odds of severe symptoms but improvement in severity scores (-0.04‒-0.01/year, p-values<0.05) and the odds for mild fever and chills, excessive fatigue, headache, and throat symptoms (0.65–0.79/year, p-values<0.05). Our study suggests that after the onset of respiratory and severe non-respiratory symptoms associated with dampness/mold, remediation efforts might not be effective in improving occupants’ health.

## Introduction

Indoor dampness and mold are public health hazards for various respiratory and non-respiratory illnesses [1–3]. The World Health Organization reported that the prevalence of indoor dampness is high but widely variable from 10 to 50% depending on countries, continents, and climate zones [3]. Mudarri and Fisk estimated that dampness and mold in homes accounted for 21% of current asthma in the United States, which costs 3.5 billion dollars annually [4]. They also reported that asthma risks from such exposure in schools, offices, and institutional buildings are similar to those in homes.

Yet, intervention studies of occupants in moisture-damaged buildings are limited. The Cochrane Collaboration reviewed eight publications that fulfilled its inclusion criteria, along with four academic dissertations, and published their findings in 2011 [5]. The reviewed studies were of low to moderate quality and they found limited evidence that water damage remediation of residential and office buildings might decrease chest symptoms and respiratory infections. And they could not generalize the findings to make a solid conclusion because of small study populations and no adjustment for potential confounding factors in those studies.

In our study, we collected up to four repeated measurements on the respiratory and non-respiratory health, and demographics of 1,447 occupants, as well as microbial levels in floor dust, from selected workstations in a large office building over seven years. Using these repeated health and environmental measurements, we examined changes in occupants’ health over time after accounting for associations with microbial exposures.

## Background

**Fig 1:**
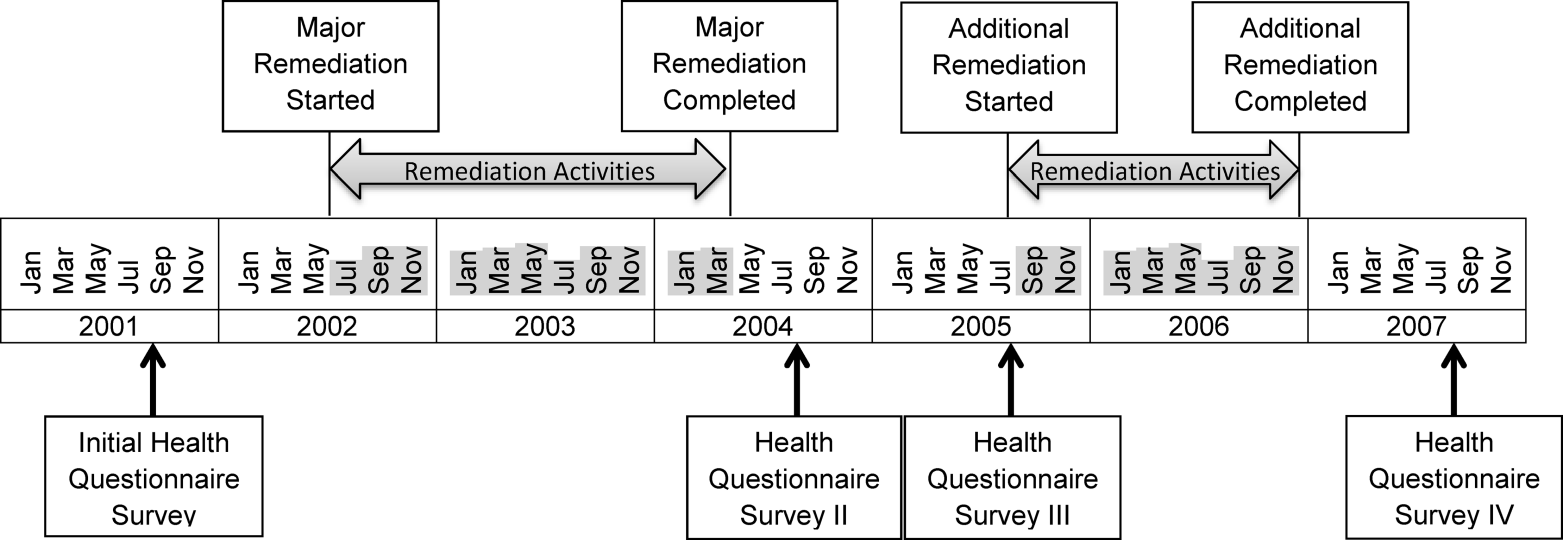
Timeline of the four health questionnaire surveys including environmental sampling and remediation. The shaded months indicate the time of remediation activities.

The study building had a long history of water incursion since being built in 1985. The current tenants had occupied the building since 1994 until they were relocated starting in 2014. Due to persistent water incursion through exterior walls, roofs, and around terraces and windows on the upper floors, building-wide major remediation was implemented between late 2002 and early 2004 and additional remediation was carried out in 2006 (Fig 1). Detailed information on the building and remediation has been published previously [6]. Building occupants had reported respiratory symptoms that they perceived to be building-related within a few months of occupancy in 1994. In previous publications using initial survey data, we reported 67 cases of post-occupancy-onset asthma, eight of hypersensitivity pneumonitis, and six of sarcoidosis. We also documented a 7.5-fold increase in new-onset asthma among occupants after building occupancy [7]. In addition, we reported that fungi and endotoxin in floor dust were a risk factor for physician-diagnosed post-occupancy-onset asthma, as well as various respiratory and non-respiratory symptoms [8,9].

## Methods

### Study and remediation timeline and study population

We conducted four cross-sectional surveys of occupants in a 20-story office building in the northeastern United States. The initial health and environmental surveys were conducted in 2001 and 2002, and two subsequent surveys in 2004 and 2005, 5 months and 17 months after major remediation, respectively, that was undertaken between late 2002 and early 2004. A final survey was conducted in 2007, 8 months after completion of additional remediation between late 2005 and 2006 (Fig 1). The building had an average of 1,240 employees over the survey period. In our longitudinal analyses, we included the 1,175 employees who had occupied the building before the initial health survey in September 2001 (long-tenured employees) and participated in at least one of the four surveys. We also analyzed 169 employees who newly occupied the building in January 2004 or later (new employees).

### Health and environmental surveys

We invited all occupants to participate in self-administered questionnaire surveys. Consent was informed on the first page of the questionnaire and it indicated that consent to participate was implied by completing the questionnaire, as approved by the National Institute for Occupational Safety and Health (NIOSH) Institutional Review Board. All surveys included questions on respiratory and non-respiratory symptoms (mucous membrane irritation or systemic symptoms) occurring in the last 12 months and at least once weekly in the last 4 weeks, building-relatedness of the symptoms, medical diagnoses, demographic and smoking information, and work history. The respiratory symptoms included wheezing, chest tightness, attacks of shortness of breath (SOB), coughing attacks, awakened by breathing difficulty, SOB on exertion, nasal symptoms (stuffy, itchy or runny nose), and sinus symptoms (sinusitis or sinus problems). The non-respiratory symptoms included throat symptoms (hoarseness or a dry, sore, or burning throat), eye symptoms (watery or itchy eyes), episodes of flu-like achiness or achy joints, episodes of fever and chills, excessive fatigue, headache, concentration difficulty, and a rash or itchy skin. We defined building-related symptoms as those improving when away from the building (i.e., over the weekend or during a holiday or vacation).

For each environmental survey, we collected floor dust from selected workstations by vacuuming carpeted floor using Backpack vacuum samplers (Pro-Team Inc., Boise, ID, USA). The dust sample aliquots were analyzed for culturable fungi, ergosterol using gas-chromatography tandem mass spectrometry, and endotoxin using *Limulus* amoebocyte lysate assay. More detailed information on sampling and analytical methods has been described elsewhere [6,8,10,11].

### Statistical analyses

For analyses of severity scores, we categorized the participants into three groups–a ‘severe symptom’ group for those who reported building-related symptoms occurring one or more times every week in the last 4 weeks, a ‘mild symptom’ group for those who reported building-related symptoms occurring in the last 12 months but not one or more times every week in the last 4 weeks, and a ‘no symptom’ group. Severity score variables based on each symptom were coded “2” for severe symptom, “1” for mild symptom, and “0” for no symptom.

We used random intercept mixed-effect regression models to examine changes in the odds of building-related respiratory and non-respiratory symptoms (binary outcome) and severity scores (continuous outcome) between two consecutive surveys (categorical variable) or for all four surveys (continuous variable in trend analyses and categorical variable for all other analyses). For binary outcomes, the models were specified with the logit link function and binary distribution. For continuous outcomes, the linear mixed models were specified with the identity link function and normal distribution, which provides a robust maximum likelihood estimator of fixed effects even when the error distribution is misspecfied [12]. The regression models were adjusted for race and sex as time-invariant covariates and smoking status; age; building tenure; and loads of fungi (colony forming unit/m^2^ of floor area), ergosterol (ng/m^2^), and endotoxin (endotoxin unit/m^2^) as time-varying covariates. We assigned the floor average of microbial load as an exposure to those who occupied the same floor from which the dust samples were collected in each survey.

In analyses to examine the time trend in odds or severity scores across the survey period, the survey time as a continuous variable was coded as 1 for 2001, 4 for 2004, 5 for 2005, and 7 for 2007. In these trend analyses, we modeled mild symptoms (versus no symptom) and severe symptoms (versus non-severe or no symptom) separately, as well as combined (mild or severe) symptoms (versus no symptom). To estimate regression parameters, we used the restricted maximum likelihood estimation method. Using the mixed models with the same specification above, we also examined if the odds of building-related severe symptoms after the major remediation were significantly different between long-tenured employees and new employees and if there were significant interaction effects between the tenure status and survey year (2005 and 2007). All statistical analyses were performed using SAS 9.2 (SAS Institute Inc., Cary, NC, USA) and statistical significance was set as a p-value ≤ 0.05 and marginal significance at 0.05 < p-value ≤ 0.1.

## Results

The participation in the health questionnaire surveys ranged from 60 to 67%. The majority of the 1,175 long-tenured employees included in the analyses were white, and 57% were females (Table 1). At the time of the initial survey, participants had worked in the building for an average of 5.9 years with a mean age of 45.7 years. More than half had never smoked and only 13% were current smokers.

**Table 1:** Characteristics of study population (n = 1,175) who participated in any one of the four surveys and occupied the building before the initial 2001 survey.

Characteristics (at the time of the initial survey)		Number or year (% or standard deviation, SD)
Race		
	White	823 (70.0)
	Black	201 (17.1)
	Others	113 (9.6)
	Missing	38 (3.2)
Sex		
	Female	674 (57.4)
	Male	499 (42.5)
	Missing	2 (0.2)
Average age, year (SD)		45.7 (8.5)
Average building tenure, year (SD) SD		5.9 (2.0)
Smoking status		
	Never	716 (60.9)
	Former	301 (25.6)
	Current	154 (13.1)
	Missing	4 (0.3)

**Fig 2:**
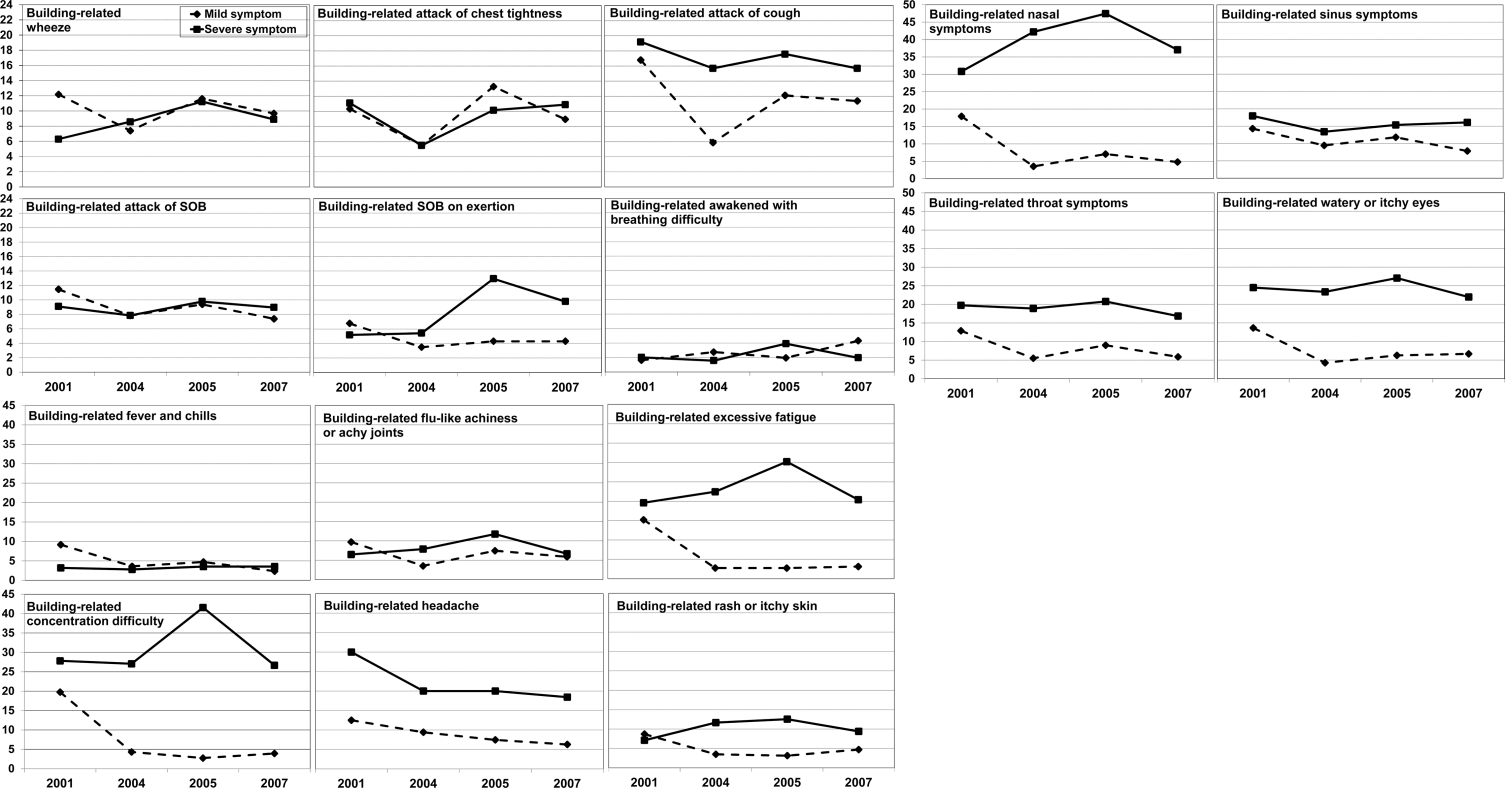
Prevalence (%) of building-related respiratory and non-respiratory mild and severe symptoms. Prevalence was based on employees who occupied the building before the initial survey and participated in at least one survey.

Prevalences of severe symptoms over the four surveys were always higher than mild symptoms for attacks of cough, upper respiratory symptoms, eye and throat symptoms, excessive fatigue, difficulty concentrating, and headache and usually higher than mild symptoms for all other symptoms (Fig 2). Prevalences of severe nasal symptoms, difficulty concentrating, and excessive fatigue were much higher than those of any other severe symptom. Overall, prevalences of lower respiratory symptoms did not constantly decline over time. Most of the severe and mild lower respiratory symptom prevalences were lower in 2004 and 2007 after the remediation compared to those in the initial and 2005 surveys, respectively. However, severe wheeze, chest tightness, and SOB on exertion, as well as mild symptom of awakening with breathing difficulty were exceptions. Similar to lower respiratory symptoms, prevalences of severe upper respiratory, eye, throat, and systemic symptoms did not decline over the study period. We found similar results when we limited the analyses to the 258 employees who participated in all four surveys (data not shown).

### Changes in lower and upper respiratory symptoms

Table 2 shows changes in the odds of building-related severe symptoms and severity scores between two consecutive surveys with or without remediation. The major remediation activities between late 2002 and early 2004 did not significantly decrease the odds of severe respiratory symptoms in the 2004 post-remediation survey compared to those in 2001. Indeed, wheezing and nasal symptoms became more prevalent (OR = 1.63 and 2.27, respectively, p-values<0.05) and nasal symptoms became more severe (increase in severity score = 0.15, p<0.05) after remediation activity.

**Table 2:** Changes in the odds of building-related severe symptoms and the severity scores between two consecutive surveys analyzed with generalized linear mixed models.

Building-related symptoms	Changes in odds and severity score between two consecutive surveys (earlier survey is the reference year)					
	2001 to 2004 (Remediation)		2004 to 2005 (No remediation)		2005 to 2007 (Remediation)	
	OR (95% CI)	Severity (SE)	OR (95% CI)	Severity (SE)	OR (95% CI)	Severity (SE)
Lower respiratory symptoms						
Wheezing	1.63 (1.00‒2.67)**	0.03 (0.04)	1.46 (1.02‒2.09)**	0.12 (0.03)**	0.84 (0.54‒1.28)	-0.04 (0.04)
Chest tightness	0.70 (0.44‒1.12)	-0.14 (0.04)**	1.85 (1.28‒2.69)**	0.18 (0.03)**	0.66 (0.43‒1.02)*	-0.12 (0.04)**
Attacks of shortness of breath	1.13 (0.70‒1.80)	-0.04 (0.04)	1.19 (0.81‒1.74)	0.06 (0.03)**	0.87 (0.55‒1.37)	-0.03 (0.04)
Cough attacks	1.35 (0.93‒1.97)	-0.05 (0.05)	1.12 (0.82‒1.53)	0.11**(0.04)	0.85 (0.58‒1.24)	-0.05 (0.05)
Awakened by breathing difficulty	0.51 (0.19‒1.39)	-0.03 (0.02)	1.60 (0.84‒3.04)	0.04**(0.02)	0.57 (0.27‒1.20)	-0.03 (0.03)
Shortness of breath on exertion	1.58 (0.98‒2.55)*	0.02 (0.04)	1.69 (1.16‒2.45)**	0.12**(0.03)	0.74 (0.48‒1.14)	-0.04 (0.04)
Nasal and Sinus symptoms						
Nasal symptoms	2.27 (1.67‒3.08)**	0.15 (0.06)**	0.95 (0.73‒1.22)	0.03 (0.04)	0.71 (0.52‒0.96)**	-0.16 (0.06)**
Sinus problems	1.05 (0.73‒1.52)	-0.08 (0.05)	0.99 (0.72‒1.38)	0.04 (0.03)	1.12 (0.75‒1.68)	0.03 (0.04)
Mucous membrane and systemic symptoms						
Throat symptoms	1.15 (0.81‒1.64)	-0.10 (0.05)**	1.14 (0.84‒1.54)	0.08 (0.04)**	0.64 (0.44‒0.92)**	-0.15 (0.05)**
Eye symptoms	1.03 (0.75‒1.42)	-0.12 (0.05)**	1.26 (0.95‒1.66)	0.11 (0.04)**	0.82 (0.58‒1.15)	-0.06 (0.05)
Flu-like achiness	0.98 (0.60‒1.61)	-0.08 (0.04)**	1.24 (0.84‒1.83)	0.08 (0.03)**	0.64 (0.39‒1.04)*	-0.08 (0.04)**
Fever and chills	0.83 (0.43‒1.59)	-0.08 (0.03)**	1.08 (0.60‒1.93)	0.01 (0.02)	0.92 (0.43‒1.97)	-0.005 (0.02)
Excessive fatigue	1.16 (0.81‒1.65)	-0.08 (0.05)	1.23 (0.93‒1.63)	0.08 (0.04)*	0.51 (0.36‒0.72)**	-0.22 (0.05)**
Headache	1.00 (0.72‒1.38)	-0.11 (0.05)**	0.90 (0.67‒1.21)	-0.02 (0.04)	0.76 (0.53‒1.09)	-0.09 (0.05)*
Concentration difficulty	1.29 (0.94‒1.77)	-0.07 (0.06)	1.52 (1.17‒1.97)**	0.19 (0.05)**	0.63 (0.46‒0.86)**	-0.19 (0.06)**
Rash or itchy skin	1.46 (0.91‒2.34)	0.01 (0.04)	1.13 (0.78‒1.65)	0.04 (0.03)	0.66 (0.41‒1.05)*	-0.07 (0.04)*

All models were adjusted for race; sex; smoking status; age; building tenure; and loads of endotoxin, ergosterol, and culturable fungi in floor dust. The earlier survey year is the reference year. OR: Odds ratio; 95% CI: 95% confidence interval; SE: standard error

Positive severity score indicates worsening in severity compared to the reference year

Negative severity score change: improvement in severity compared to the reference year

*: 0.05<p-value≤ 0.1

**: p-value≤0.05.

During the period of no remediation between the 2004 and 2005 surveys, the odds of wheezing, chest tightness, and SOB on exertion in 2005 significantly increased (OR = 1.46‒1.85, p-values<0.05) and all lower respiratory symptoms became more severe (increase in severity score = 0.04‒0.18, p-values<0.05) than those in 2004 although upper respiratory symptoms did not change. When we compared respiratory symptoms in 2005 (17 months after the major remediation activity) to those in the 2001 initial survey we also found significant worsening in the odds (OR = 2.08‒3.00; p-values<0.001) for severe wheezing, SOB on exertion, and nasal symptoms and their severity (severity score = 0.15‒0.17, p-values<0.05) (data not shown). Likewise, additional remediation activity after the 2005 survey did not significantly improve the odds of severe respiratory symptoms or decrease the symptom severity in 2007, except for chest tightness that was marginally improved in odds (OR = 0.66, p = 0.058) and significantly decreased in severity (severity score = -0.12, p<0.05) and nasal symptoms that were significantly (p-values<0.05) improved in odds (OR = 0.71) and decreased in severity (score = -0.16).

**Fig 3:**
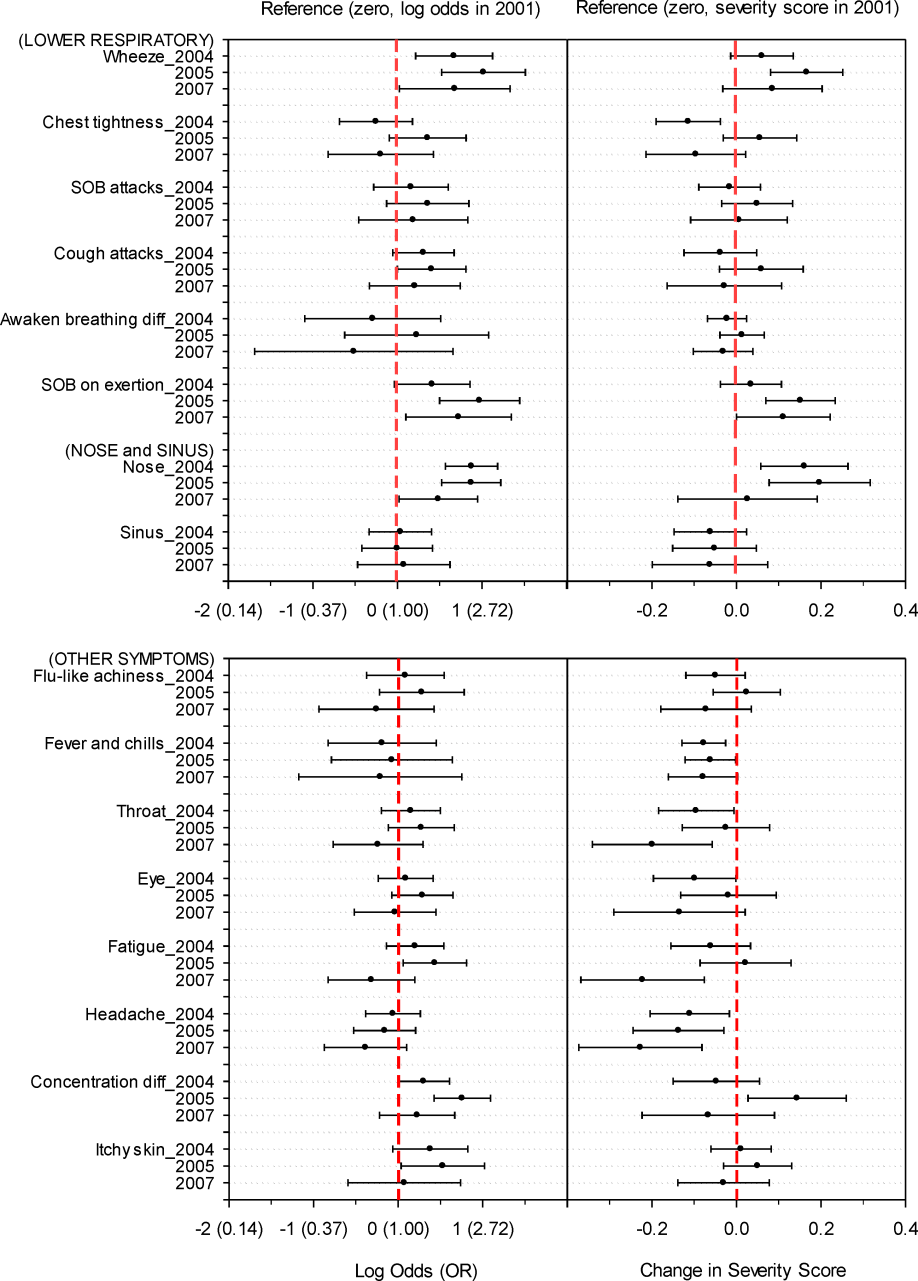
Longitudinal changes in odds of building-related severe symptoms and severity scores. Generalized linear mixed models were used with the survey year as a categorical variable. The vertical solid lines in each panel are odds [log odds = 0 (OR = 1.0)] or severity score (set as zero change) of the 2001 survey as a reference.

Fig 3 compares the odds of severe symptoms and severity scores in the three follow-up surveys to those in the initial survey. Odds of severe respiratory symptoms (wheeze, SOB attacks, cough attacks, SOB on exertion, and nasal symptoms) in the follow-up surveys did not drop below those in the initial survey. The severity scores of wheeze, SOB on exertion, and nasal symptoms during the follow-up surveys were higher than the initial survey.

When we analyzed symptoms in the last 12 months that combines mild and severe symptoms using linear trend analyses (Table 3), there were no significant changes in the odds of any respiratory symptom over time, except for sinus problems. However, when we analyzed severe symptoms and mild symptoms separately, we found that odds of severe wheezing, SOB on exertion, and nasal symptoms showed a significantly increasing trend (12 to 16% increase in odds per year; p-values < 0.05) and that the symptom severity worsened over time. However, the odds of mild wheezing, SOB on exertion, and nasal symptoms generally decreased over the study period although they did not reach statistical significance, except for nasal symptoms. For all other respiratory symptoms, we did not find any significant changes in odds of severe symptoms or severity scores over the study period. Therefore, despite general declines in the odds of mild respiratory symptoms over time, there were significant increases or no significant changes in the symptom severity scores as well as the odds of severe symptoms. This indicates that more occupants in the mild respiratory symptom group (especially, wheezing, SOB on exertion, and nasal symptoms) tended to move to the severe symptom group over time than to the asymptomatic group. However, for sinus symptoms, more occupants in the mild symptom group seemed to move to the asymptomatic group than to the severe symptom group. When we performed the same trend analysis for severe respiratory symptoms using asymptomatic participants as a comparison group, we obtained similar results.

**Table 3:** Trend analysis on changes in the odds of building-related symptoms and severity scores over 7 years of the survey period using generalized linear mixed models.

Symptoms	Odds ratio (95% CI) per year			Severity score per year (SE)
	Combined (severe/mild) symptom	Severe symptom only	Mild symptom only	
Lower respiratory symptoms				
Wheezing	1.06 (0.97‒1.15)	1.15 (1.04‒1.28)**	0.95 (0.86‒1.06)	0.02 (0.01)**
Chest tightness	0.93 (0.86‒1.01)	1.00 (0.91‒1.11)	0.89 (0.80‒0.99)**	-0.01 (0.01)
Attacks of SOB	0.99 (0.91‒1.08)	1.05 (0.94‒1.16)	0.95 (0.85‒1.06)	0.004 (0.01)
Cough attacks	0.97 (0.90‒1.05)	1.05 (0.97‒1.15)	0.91 (0.83‒1.00)*	0.0003 (0.01)
Awakened by breathing difficulty	0.95 (0.83‒1.08)	0.95 (0.79‒1.15)	0.94 (0.80‒1.11)	-0.003 (0.01)
SOB on exertion	1.07 (0.98‒1.17)	1.16 (1.05‒1.28)**	0.92 (0.81‒1.06)	0.02 (0.01)**
Nasal and Sinus symptoms				
Nasal symptoms	0.96 (0.89‒1.04)	1.12 (1.04‒1.21)**	0.75 (0.68‒0.84)**	0.02 (0.01)
Sinus problems	0.92 (0.86‒1.00)**	1.01 (0.92‒1.10)	0.86 (0.78‒0.95)**	-0.01 (0.01)
Mucous membrane and systemic symptoms				
Throat symptoms	0.86 (0.79‒0.93)**	1.00 (0.91‒1.08)	0.71 (0.63‒0.80)**	-0.03 (0.01)**
Eye symptoms	0.90 (0.84‒0.97)**	1.02 (0.94‒1.10)	0.74 (0.66‒0.83)**	-0.02 (0.01)
Flu-like achiness	0.94 (0.86‒1.02)	0.99 (0.89‒1.10)	0.90 (0.80‒1.01)*	-0.01 (0.01)
Fever and chills	0.83 (0.75‒0.93)**	0.97 (0.83‒1.13)	0.76 (0.66‒0.87)**	-0.01 (0.01)**
Excessive fatigue	0.87 (0.80‒-0.94)**	0.99 (0.92‒1.08)	0.65 (0.57‒0.74)**	-0.03 (0.01)**
Headache	0.85 (0.79‒0.92)**	0.95 (0.88‒1.02)	0.79 (0.72‒0.88)**	-0.04 (0.01)**
Concentration difficulty	0.93 (0.87‒1.00)**	1.08 (1.08‒1.16)**	0.70 (0.63‒0.79)**	0.001 (0.01)
Rash or itchy skin	0.95 (0.86‒1.04)	1.05 (0.94‒1.16)	0.81 (0.70‒0.93)**	-0.0004 (0.01)

All models were adjusted for race; sex; smoking status; age; building tenure; and loads of endotoxin, ergosterol, and culturable fungi in floor dust. 95% CI: 95% confidence interval; SE: standard error

Positive severity score change: worsening in severity compared to reference

Negative severity score change: improvement in severity compared to reference

*: 0.05<p-value≤ 0.1

**: p-value≤0.05.

### Changes in mucous membrane irritation and systemic symptoms

The odds of severe non-respiratory symptoms did not significantly decrease between 2001 and 2004, but the severity of mucous membrane irritation (throat and eye symptoms) and some systemic symptoms (flu-like achiness, fever and chills, and headache) significantly (p-values<0.05) decreased (Table 2). During the period of no remediation between the 2004 and 2005 surveys, there were no changes in the odds for the severe non-respiratory symptoms, except for difficulty concentrating [the odds in 2005 significantly increased (OR = 1.52, p-values<0.05) compared to 2004]. On the other hand, the severity scores of mucous membrane irritation and some systemic symptoms significantly increased (0.08‒0.19, p-values<0.05) during the period of no remediation activity. When we compared 2005 symptoms (17 months after the major remediation activity) to those in the 2001 initial survey we found no significant changes in the odds of most of the non-respiratory symptoms, except for a significant increase in the odds of excessive fatigue and difficulty concentrating (OR = 1.59‒1.90, p-values<0.05); however, there were no changes in the severity for all non-respiratory symptoms, except for improvement in headaches (severity = -0.14, p<0.05). Additional remediation after the 2005 survey significantly decreased the odds of severe throat and some systemic symptoms (OR = 0.51‒0.64, p-values<0.05) and decreased the symptom severity (severity scores = -0.22‒-0.08, p-values<0.05). In contrast to respiratory symptoms, the severity of mucous membrane irritation and systemic symptoms among the follow-up surveys had improved at least once over the survey period compared to the initial survey, except for flu-like achiness, difficulty concentrating, and skin symptoms (Fig 3).

Trend analyses of combined symptoms (Table 3) showed that the odds of most non-respiratory symptoms tended to significantly decrease over time (7 to 17% per year; p-values<0.05). In addition, the severity of throat symptoms and some systemic symptoms (fever and chills, excessive fatigue, and headache) showed a significantly improving trend over the study period. However, when we analyzed severe and mild symptoms separately, the odds of all severe non-respiratory symptoms, except for difficulty concentrating, did not change over time but the odds of all mild non-respiratory symptoms significantly decreased. When we performed the same trend analysis for severe non-respiratory symptoms using asymptomatic participants as a comparison group, we obtained similar results. Taken together, our results indicate that the improving trend in mucous membrane irritation and systemic symptoms over the study period generally resulted from improvement of occupants in the mild symptom group.

### Odds of symptoms in long-tenured versus new employees

**Fig 4:**
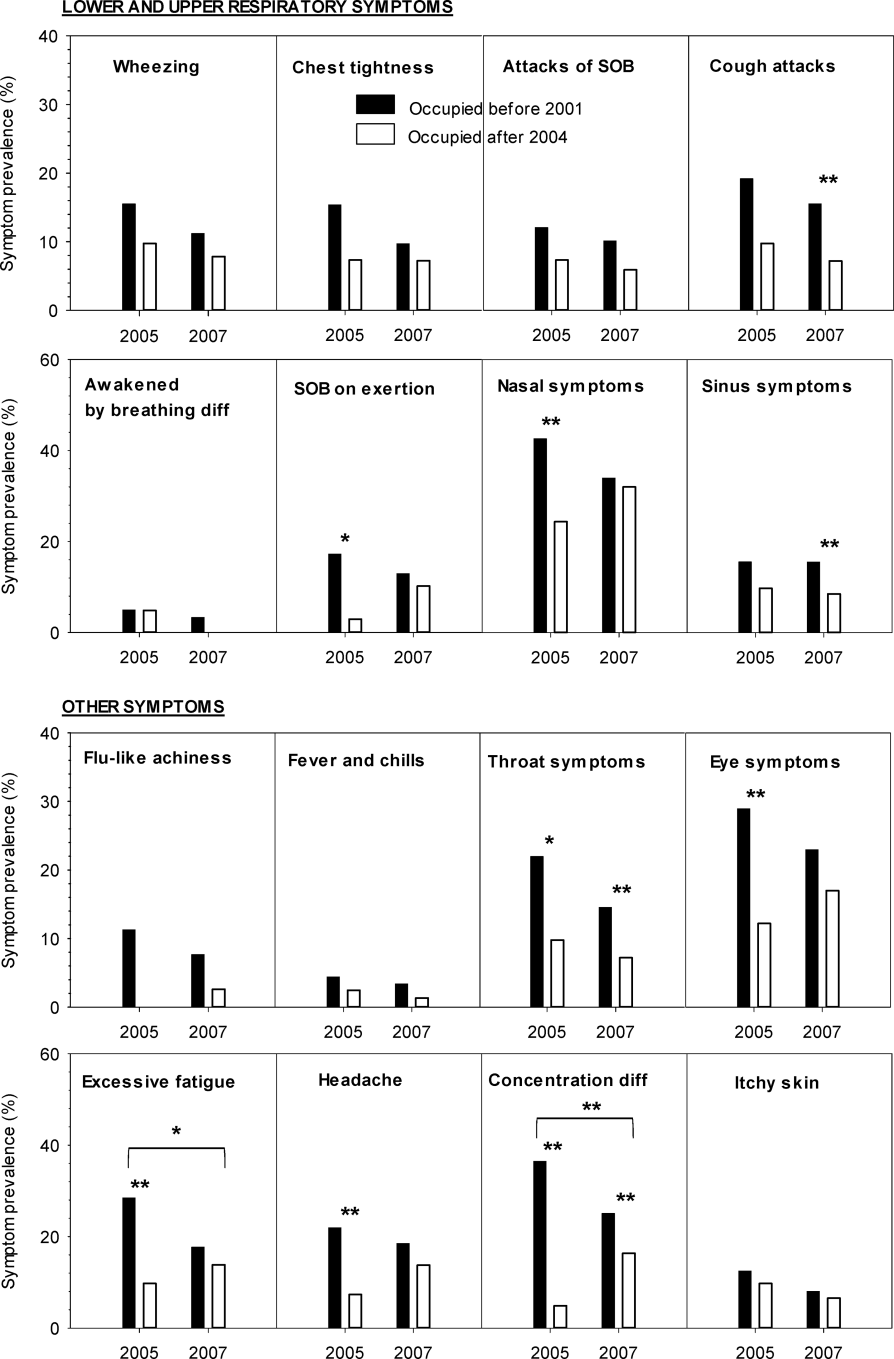
Prevalence (%) of building-related severe symptoms by tenure status and survey year. Tenure status: occupied before 2001 survey versus January 2004 or later; and survey year: 2005 versus 2007. Marginal or significant interaction effects between tenure status and survey year. *0.05<p-value≤0.1; **p-value≤0.05.

We compared the odds of severe symptoms in long-tenured employees to those in new employees who were hired after completion of the major remediation (n = 169) (Fig 4). The odds of lower respiratory and some systemic symptoms in the new employees were consistently lower than those in long-tenured employees both in 2005 and 2007, but they did not reach statistical significance, except for attacks of cough and SOB on exertion. The odds of nasal symptoms was lower in 2007 than 2005 in long-tenured employees while it was reversed in new employees. We found similar reversed trends for SOB on exertion, and all systemic (except for fever and chills) and eye symptoms in 2005 and 2007 in the two tenure groups. There were significant or marginally significant interactions between the tenure group and the survey year for difficulty concentrating and excessive fatigue, respectively.

## Discussion

We found from our longitudinal analyses taking the effect of microbial exposures into account over a seven year period that: 1) once building-related respiratory symptoms (asthma and nasal symptoms) had developed, they did not generally improve despite the various remediation activities; 2) building-related severe non-respiratory (mucous membrane irritation and systemic) symptoms also did not improve after the remediation activities; 3) however, building-related mild non-respiratory symptoms tended to improve over time. In our analyses of 1,175 office employees, we also took into account within-subject variability of up to four repeated measurements of their health over the study period. Our study findings agree with other remediation studies conducted in Europe. Follow-up studies of teachers immediately and two years after remediation of schools in Finland reported no significant remediation effects on respiratory symptoms and spirometry test results, although there were significant decreases in respiratory infections and no new asthma diagnoses [13]. Rudblad and colleagues [14] conducted four repeated measurement studies (one before and three after remediation) of school teachers in one index school and one control school in Sweden. They found that nasal mucosal hyperreactivity measured with rhinostereometry was persistent even three years after remediation.

Our previous report [15] documented that new cases of post-occupancy physician-diagnosed asthma and hypersensitivity pneumonitis had declined every year since 2001 among the same building population. Our current analyses also show that new employees hired after the major remediation had generally lower odds of severe respiratory and non-respiratory symptoms. In addition, we previously documented from analyses of 97 employees (a subset of the current study population) who participated in both the 2002 and 2005 medical examinations, that their spirometry test results and medication use did not indicate overall improvement in their respiratory health [16]. We have also demonstrated [17] that those who had already developed building-related rhinosinusitis symptoms in the initial survey among long-tenured employees had more than a two-fold increase in risk of developing building-related asthma and asthma symptoms in the follow-up surveys. This increased risk was independent of exposure to endotoxin and fungi since the statistical models were adjusted for these exposures. All of these results are consistent with our current findings that remediation activities might decrease the risk of developing new respiratory cases among new employees, but these remediation activities often do not improve existing respiratory and severe non-respiratory symptoms among occupants who had been affected by moisture-damaged building environments for an extended period of time.

Haverinen-Shaughnessy et al. monitored and evaluated remediation processes for seven different buildings and concluded that achieving successful remediation was challenging [18]. Our study building had undergone building-wide and extensive major remediation between 2002 and 2004, including roof replacement, repair of window flashing, repair of exterior walls and balconies, and replacement of damaged carpets and wallboards. Additional remediation was carried out after the 2005 survey, including repair of exterior walls around windows, and replacement of carpets, wallboards, and ceiling tiles on the upper floors [6]. Our previous publication on the remediation effect on microbial levels in floor dust in this building showed that the major remediation in 2003 remained effective until two years after its completion, although this effect was not sustained in 2007, implying that the major remediation might have been only partially successful [6]. Thus, we cannot exclude the possibility that the lack of remediation effects on occupants’ health in our study might have resulted from persistent exposures in the building even after the various remediation activities. However, because we adjusted our statistical models for microbial exposures to endotoxin, ergosterol, and culturable fungi as time-varying covariates that were significant risk factors for various respiratory and non-respiratory symptoms in the same population [19], the potential confounding effects of these persistent exposures were likely to be minimized. A potential explanation of our findings might be that once respiratory or severe non-respiratory symptoms have developed in occupants of moisture-damaged buildings, physiological recovery in the affected employees might be compromised. Thus, our findings provide important information on the natural history of occupants’ health in relation to remediation activity.

Jarvis and Morey [20] reported that remediation, compared to the baseline data from a damaged building, significantly improved respiratory and non-respiratory symptom odds among occupants who reoccupied the remediated index building from a relocated building after approximately 3.5 years. However, when they compared lower respiratory symptom odds in the same occupants before relocation and several months after the relocation, they did not find significant changes. They suggested that recovery might be a long process after remediation and removal from exposure, which is also consistent with Rudblad and colleagues’ observation discussed earlier [14]. Thus, another potential explanation for no improvement in respiratory and severe non-respiratory symptoms over the study period could be that recovery might require more time than our follow-up studies allowed (3.5 years after major remediation).

On the other hand, a randomized trial study on asthmatic children aged 2–17 years old in moldy homes in Ohio, U.S. [21] showed that the remediation group (n = 29) had significantly decreased symptom days and health care use compared to the control group (n = 33). Patovirta and colleagues [22] also found from three school studies conducted before and after moisture and mold remediation that non-respiratory symptoms (fatigue and headache) in school teachers were significantly reduced, but upper respiratory symptoms (allergic rhinitis), sinusitis, and conjunctivitis were persistent or even higher after the remediation. Ebbehøj and colleagues [23] also observed decreased building-related non-specific symptoms (mucous membrane irritation and systemic symptoms) in occupants after remediation and thorough cleaning. However, none of these studies have examined mild and severe non-respiratory symptoms separately or changes in the symptom severity over time. Trend analyses in our study showed that severity scores of non-respiratory (eye, throat, and systemic) symptoms significantly improved during the study period; however, these improvements were driven by improvement in the mild symptom group only. These findings indicate that, in contrast to respiratory symptoms or severe non-respiratory symptoms (mucous membrane irritation and systemic symptoms), mild non-respiratory symptoms might be improved over time if remediation is completed before these symptoms become severe.

One of strengths in our study is that the findings are based on longitudinal analyses of 1,175 occupants that accounted for temporal variance of health outcomes within the same occupant. In addition, all our statistical models were adjusted for race and sex as time-invariant covariates, and age; smoking status; building tenure; and environmental exposures of fungi, ergosterol, and endotoxin as time-varying covariates. Thus, our study overcomes limitations of small sample sizes and a lack of controlling confounding factors in other published studies that were identified by the 2011 Cochrane Collaboration’s review [5]. On the other hand, our repeated measurement studies were not randomized controlled trials but rather were observational studies, and thus, remediation activities could not be blinded to the building occupants. Therefore, occupants’ recognition of remediation might have resulted in bias in reporting their symptoms in 2005 and 2007 toward false positive remediation effects. However, this bias does not explain our findings of improvement in mild non-respiratory symptoms because the bias cannot selectively occur only in the mild non-respiratory symptom group.

## Conclusions

Our study indicates that once respiratory or severe non-respiratory symptoms have developed from long-term exposure to dampness and mold, the symptoms might not be easily improved despite various remediation activities. Our findings suggest that in moisture-damaged buildings with sentinel cases of building-related lung disease, the best public actions would be prompt relocation of affected employees, which might prevent further exacerbation of their illness or prompt remediation once water leaks are identified, that is before respiratory and severe non-respiratory symptoms have developed in building occupants.
